# Proteomic dataset of knockdown of AKT3 on protein expression and function in female germline stem cells

**DOI:** 10.1016/j.dib.2024.111112

**Published:** 2024-11-13

**Authors:** Yue Shen, Chunlan Mu, Qingling Jia, Xiaoyong Li, Ji Wu

**Affiliations:** a Key Laboratory of Fertility Preservation and Maintenance of Ministry of Education, School of Basic Medical Sciences, Ningxia Medical University, Yinchuan 750004, China; b Key Laboratory for the Genetics of Developmental & Neuropsychiatric Disorders (Ministry of Education), Bio-X Institutes, Shanghai Jiao Tong University, Shanghai 200240, China

**Keywords:** FGSCs, Proteomics, Pathway analysis

## Abstract

Female germline stem cells (FGSCs) are adult stem cells capable of self-renewal and differentiation into mature oocytes. AKT3, a member of the AKT kinase family, plays crucial roles in multiple cellular processes, such as proliferation, migration, and apoptosis. However, the mechanism by which AKT3 affects the development of FGSCs is poorly understood. We performed 4D-data-independent acquisition (DIA) Quantitative Proteomics on mouse FGSCs in which AKT3 was knocked down using a lentivirus and on control FGSCs. Based on the raw DIA data, coupled with database searches and data filtering, we identified 46,260 peptides, including 45,821 unique peptides, corresponding to 6849 identified proteins and 6697 comparable proteins. These identified proteins were functionally annotated using Gene Ontology (GO), Kyoto Encyclopedia of Genes and Genomes (KEGG), Protein Domain, Clusters of Orthologous Genes (COG)/Eukaryotic Orthologous Groups (KOG), STRING database, Reactome, WikiPathways, HallMark, and transcription factor (TF) analyses. Fisher's exact test was used to assess the significance of functional enrichment of the differentially abundant proteins. We identified 281 differentially abundant proteins between AKT3 knockdown and control FGSCs, comprising 229 upregulated and 52 downregulated proteins. We performed clustering analysis on these differentially abundant proteins based on functional enrichment using GO, Domain, KEGG, Reactome and WikiPathways platforms. A protein–protein interaction network was constructed to demonstrate interactions between proteins. These datasets will facilitate future investigations into the mechanisms governing FGSC self-renewal and differentiation and will provide a foundation for understanding diseases related to abnormal germ cell development.

Specifications Table

**Table:** 

Subject	Cell Biology
Specific subject area	Reproductive Physiology, Epigenetic, Proteomics
Type of data	Table, Graph, Figure. Raw, Analyzed, Filtered.
Data collection	The FGSCs with AKT3 knockdown and their respective controls were established based on lentiviral infection. Proteomic analysis of these FGSCs were performed using 4D-DIA (Data-Independent Acquisition) Quantitative Proteomics technology. The DIA data were processed using DIA-NN search engine (v.1.8). Tandem mass spectra were searched against Mus_musculus_10090_SP_20231220. fasta (17191 entries) concatenated with reverse decoy database. Further analysis was conducted using eggnog-mapper (v2.1.6), PfamScan (v1.6), and diamond (v2.0.11.149).
Data source location	Institution: Bio-X institutes, Shanghai Jiao Tong University City: Shanghai Country: China.
Data accessibility	Repository name: PRIDE Data identification number: PXD054663 Direct URL to data: http://www.ebi.ac.uk/pride/archive/projects/PXD054663 FTP Download: https://ftp.pride.ebi.ac.uk/pride/data/archive/2024/08/PXD054663
Related research article	None.

## 1 Value of the Data

• The dataset represents the differential protein abundance between control and lentivirus-mediated AKT3 knockdown FGSCs.
• The dataset presents encompass the proteomic profiles of FGSCs with AKT3 knockdown compared to controls, serving as a valuable proteomic reference for researchers aiming to explore the functions and downstream targets of AKT3.
• The dataset provides potential protein targets for studying FGSC self-renewal.

## 2 Background

Female germline stem cells (FGSCs) are a type of adult stem cells endowed with the ability to self-renewal and can differentiate into fully developed oocytes. Mammalian FGSCs were initially isolated and characterized by our laboratory [1]. Subsequently, FGSCs from rats, adult pigs, juvenile monkeys, human ovaries, and human follicular aspirates have been isolated and characterized [[2], [3], [4], [5], [6]]. AKT3, a member of the AKT kinase family, plays crucial roles in multiple cellular processes, such as proliferation, migration, and apoptosis [7,8]. It's quite fascinating to explore how AKT3 influences the proteomic changes of various genes. Here, we performed a 4D-DIA Quantitative Proteomics analysis to identify differential proteomic expression signatures of FGSCs with AKT3 knockdown and its control. This dataset reveals the role of AKT3 in FGSC development and provides a basis for follow-up studies of other genes involved in FGSC development. Findings from such studies will inform a new perspective for the prevention and treatment of female infertility and other diseases.

## 3 Data Description

**Fig. 1:**
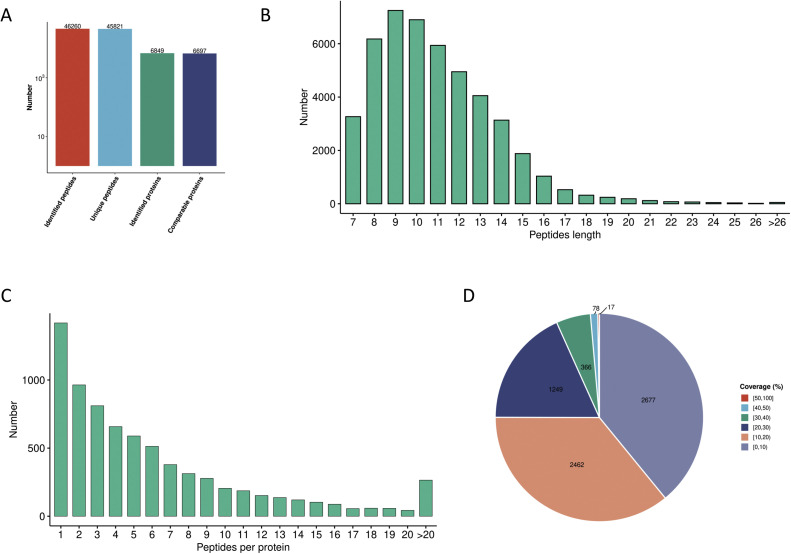
4D-DIA Quantitative Proteomics analysis of FGSCs infected with an AKT3 knockdown lentivirus and control FGSCs. (A) The number of identified peptide segments and proteins after database searches. (B) Distribution of peptide segment length. (C) Distribution of peptide segment quantity. (D) Distribution of protein coverage.

**Fig. 2:**
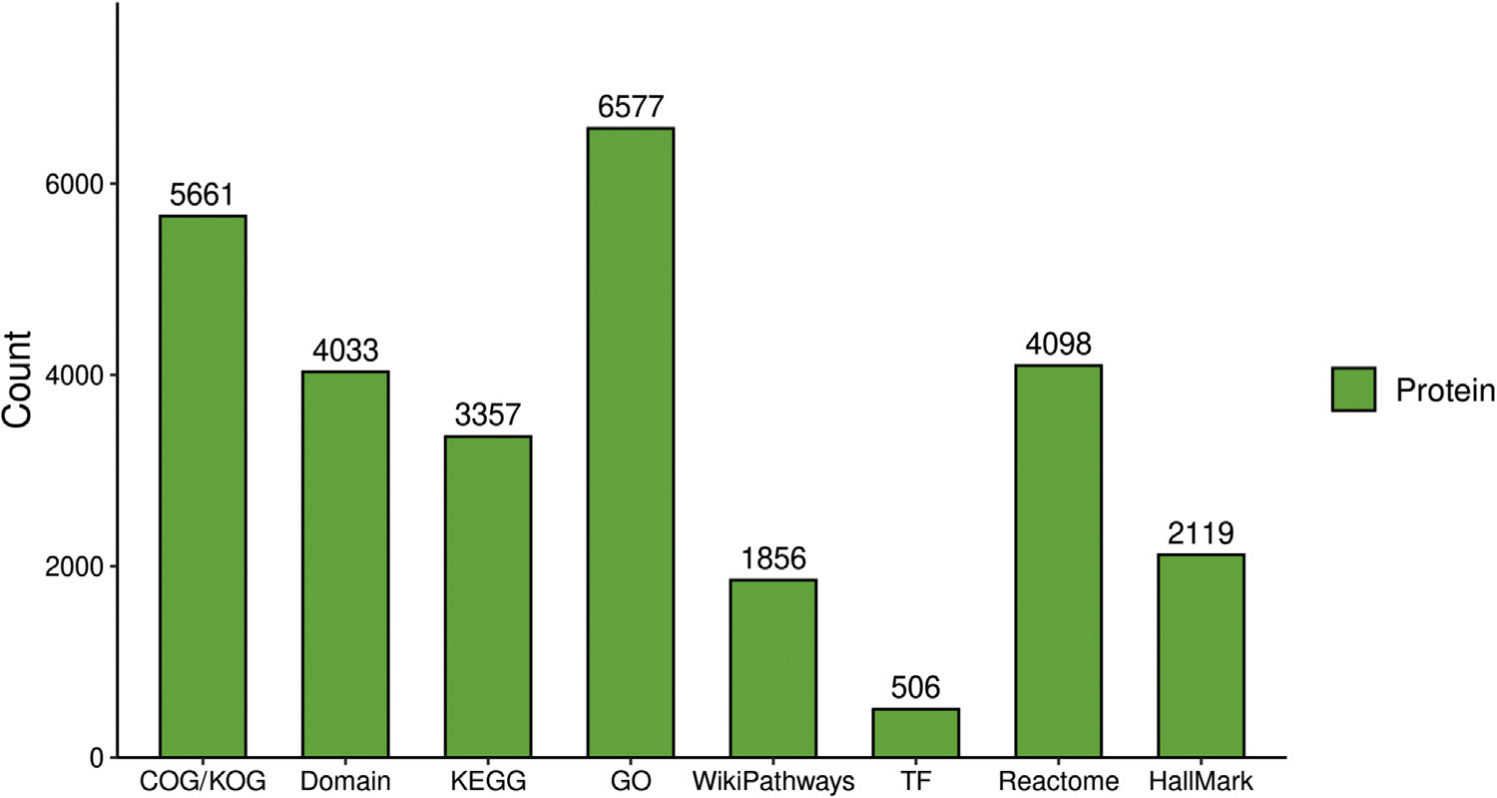
Functional annotation of identified proteins. GO, Protein Domain, KEGG Pathway, COG/KOG Functional Classification, Subcellular Localization, Reactome, WikiPathways, HallMark, and transcription factor analyses of identified proteins.

**Fig. 3:**
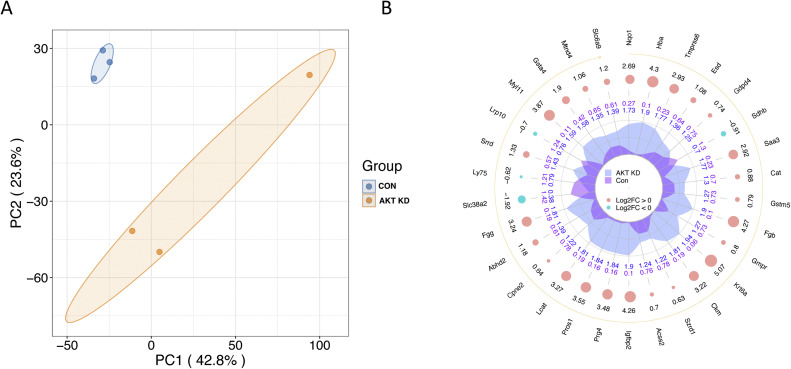
Screening for differentially abundant proteins (DAPs). (A) PCA analysis of FGSCs infected with an AKT3 knockdown lentivirus and control FGSCs. (B) Radar chart of the DAPs between FGSCs infected with the AKT3 knockdown lentivirus and control FGSCs. AKT KD, FGSCs with AKT3 knockdown; CON, control.

**Fig. 4:**
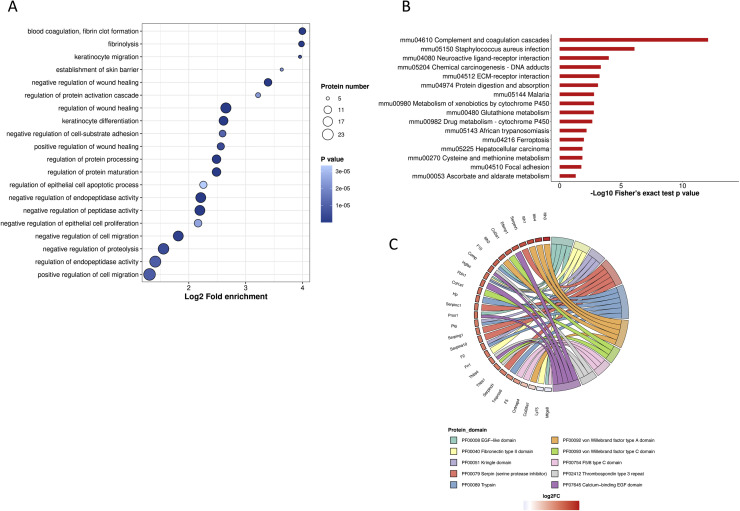
Enrichment analyses of DAPs. (A) GO enrichment analysis of DAPs. (B) KEGG pathway enrichment analysis of DAPs. (C) Protein domain analysis of DAPs.

**Fig. 5:**
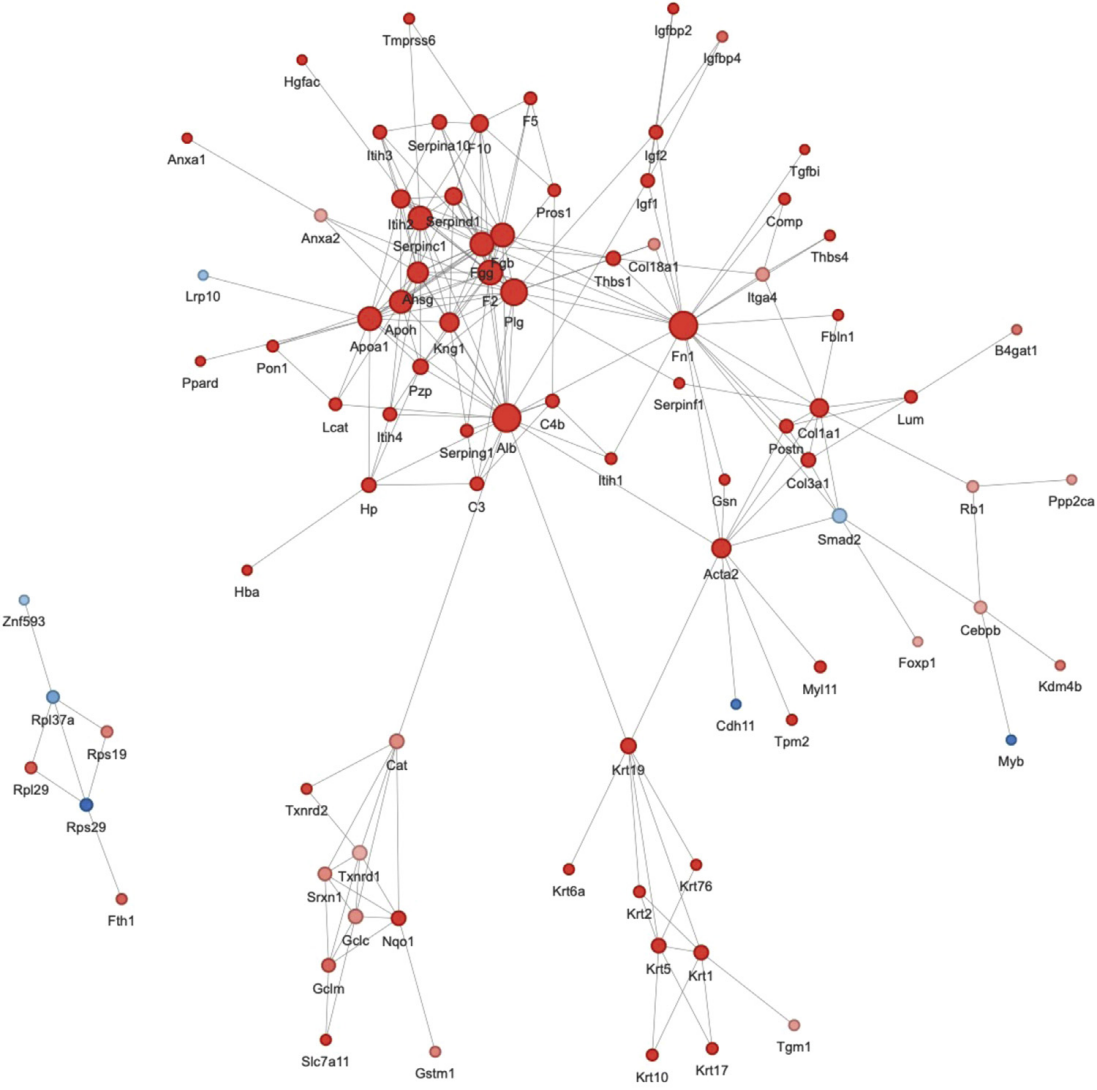
Protein–protein interaction network of DAPs between FGSCs infected with the AKT3 knockdown lentivirus and control FGSCs. The top 50 proteins with the closest interactions were selected to construct the protein–protein interaction network.

We constructed a sample-specific protein database from the raw MS files and then used software to interrogate the database. The raw data were deposited in the PRIDE repository under the identifier PXD054663. Fig. 1A depicts an overview of the identified peptide segments and protein numbers after data filtering of the search results. We then conducted a series of quality control evaluations on the data to ensure that the dataset met predefined standards, including peptide length distribution, peptide quantity distribution, and protein coverage distribution (Fig. 1B–D). To examine the functional characteristics of the identified proteins, we conducted functional annotation using GO, Protein Domain, KEGG Pathway, COG/KOG Functional Classification, Subcellular Localization, Reactome, WikiPathways, HallMark, and transcription factor (TF) analyses (Fig. 2, Table S1). A principal component analysis (PCA) plot was performed to illustrate the clustering of the samples (Fig. 3A). We identified 281 differentially abundant proteins between AKT3 knockdown and control FGSCs. These comprised 229 upregulated and 52 downregulated proteins (Fig. 3B). GO, KEGG, and domain enrichment analyses of the differentially abundant proteins were also performed (Fig. 4). To clearly display interaction between proteins, we generated a protein interaction network for the 50 proteins with the closest interactions (Fig. 5). Mass spectrometry analysis of knockdown of AKT3 on protein expression and function in FGSCs provides a data resource for future work to for understanding the mechanisms governing FGSC self-renewal and differentiation and clues for understanding diseases related to abnormal germ cell development.

## 4 Experimental Design, Materials and Methods

### 4.1 Knockdown of AKT3 in FGSCs with lentivirus

Mouse FGSCs were cultured according to previously described methods [1,9,10]. The synthesis and design of lentiviral vectors for the knockdown of AKT3 were carried out by OBiO Biotechnology (Shanghai). Cells were subjected to infection with lentiviral particles containing shRNA specific to AKT3 for a period of 24 h, in accordance with the instructions provided by the manufacturer. To identify the most efficient sequence for AKT3 knockdown, 3 distinct shRNA sequences were specifically designed to target AKT3. These shRNA sequences were then inserted into the pSLenti-U6-shRNA-CMV-EGFP-F2A-Puro-WPRE lentiviral vector, which possesses a gene for puromycin resistance. Subsequently, the cells were infected underwent selection using puromycin at a concentration of 5 μg/ml for a duration of 3 to 4 days, thus facilitating the selection of cells that exhibit a consistent and potent downregulation of AKT3.

### 4.2 Sample preparation

For 4D-DIA Quantitative Proteomics analysis, FGSCs were sonicated three times on ice with a high intensity ultrasonic processor. The cell lysate was then centrifuged at 12,000 g, 4 °C for 10 min and the protein concentration in the supernatant determined using a BCA kit. The protein solution was then treated with 5 mM dithiothreitol for 30 min at 56 °C and alkylated with 11 mM iodoacetamide for 15 min in the dark. Two hundred millimoles of TEAB were added to protein diluted to a final concentration of less than 2 M. The protein solution was then digested overnight with trypsin at a trypsin: protein ratio of 1: 50 and then for 4 h with a 1:100 trypsin: protein ratio. Finally, samples were passed through Strata X SPE columns to desalt the peptides.

### 4.3 Mass spectrometry

Tryptic peptides were solubilized in liquid chromatography mobile phase A (0.1 % formic acid, 2 % acetonitrile/in water) and separated using the NanoElute ultra-high performance liquid chromatography system. The liquid phase gradient setting was: 0–14 min, 6–24 % mobile phase B (0.1 % formic acid in acetonitrile); 14–16 min, 24–35 % B; 16–18 min, 35–80 % B; 18–20 min, 80 % B, with a flow rate maintained at 500 nl/minon. The peptide segments were separated by an ultra-high performance liquid chromatography system and then injected into a capillary ion source for ionization. Data was collected by a timsTOF Pro 2 mass spectrometer. The ion source voltage was 1.75 kV, and the peptide parent ion and its secondary fragments were detected and analyzed using the TOF method. Data collection used the data-independent parallel cumulative serial fragmentation (dia PASEF) mode, with the primary MS scanning range set to 300–1500 m/z. One primary MS collection was made, followed by 20 PASEF mode collections. The secondary MS scanning was performed in the 400–850 range, with windows every 7 m/z.

### 4.4 Database searches

The data-independent acquisition (DIA) data for this experiment were retrieved using the DIA-NN (v 1.8) search engine with default parameters. The database searched was Mus_musculus _10090 SP_20231220. fasta (17,191 sequences), with Trypsin/P as the enzyme digestion method and a maximum number of missed cuts set to 1. The fixed modification settings were N-term M exception and C carbamidomimetry. The FDR was set to < 1 %.

### 4.5 Annotation methods

For GO annotation, the GO ID of each protein was extracted using eggnog mapper software hosted by the EggNOG database (v5.0.2, http://eggnog5.embl.de/#/app/home). Protein domains were identified based on the Pfam database and PfamScan tools (A.hmm-33.1, https://www.ebi.ac.uk/interpro/entry/pfam/#table). Protein pathways were annotated based on the KEGG pathway database and BLAST alignment (blast p) was performed on the identified proteins (e-value ≤ 1e-4). For each BLAST alignment, the result with the highest annotation score was selected. WolF PSORT software was used to predict subcellular localization of annotated proteins. COG/KOG annotations were performed using eggNOG (v5.0.2, http://eggnog5.embl.de/#/app/home) and KOG (COG2020, KOG, https://ftp.ncbi.nih.gov/pub/COG/) databases. Reactome annotation, WikiPathways annotation, and HallMark's Signature Gene Sets annotation were conducted with Reactome (https://reactome.org/), WikiPathways (https://www.wikipathways.org/) and MSigDB (v2022.1, http://www.gsea-msigdb.org/gsea/msigdb/human/collections.jsp#H) databases, respectively. The public databases, TRRUST (v2, https://www.grnpedia.org/trrust/) and GTRD (v21.12, http://gtrd.biouml.org/), were used for transcription factor annotation.

### 4.6 Protein–protein interaction network

The DAPs were compared with the STRING (https://cn.string-db.org). The confidence score was set to > 0.7. The interaction network of DAPs was visualized using the package ``visNetwork'' in R.

## Limitations

Not applicable.

## Ethics Statement

Hereby, we (Yue Shen, Chunlan Mu, Qingling Jia, Xiaoyong Li, Ji Wu) have declared that we have read and follow the ethical requirements for publication in Data in Brief and confirm that the current work does not involve human subjects, animal experiments, or any data collected from social media platforms.

## CRediT Author Statement

**Yue Shen:** Formal analysis, Investigation, Methodology, Software, Validation, Visualization, Writing – original draft. **Chunlan Mu:** Data curation, Investigation, Validation. **Qingling Jia:** Investigation, Validation. **Xiaoyong Li:** Conceptualization, Funding acquisition, Resources, Supervision, Writing – original draft, Writing – review & editing. **Ji Wu:** Project administration, Conceptualization, Funding acquisition, Resources, Supervision, Writing – review & editing.

## Data Availability

PRIDEProteomics dataset of knockdown of AKT3 on protein expression and function in female germline stem cells (Original data).

## Appendix Supplementary materials

Image, application 1
